# The shared and specific mechanism of four autoimmune diseases

**DOI:** 10.18632/oncotarget.19383

**Published:** 2017-07-19

**Authors:** Meiwei Luan, Zhenwei Shang, Yanbo Teng, Xinren Chen, Mingming Zhang, Hongchao Lv, Ruijie Zhang

**Affiliations:** ^1^ College of Bioinformatics Science and Technology, Harbin Medical University, Harbin, China

**Keywords:** autoimmune diseases, share-pathway, specific-pathway, share-mechanism, specific-mechanism, Immunology

## Abstract

Interaction between genetic and epigenetic mechanisms may lead to autoimmune diseases. The features of these diseases show familial aggregation. The generality and specificity are keys to studying pathogenesis and etiology of them. This research integrated data of genetics and epigenetics, to find disease-related genes based on the levels of expression and regulation, and explored then to the shared and specific mechanism of them by analyzing shared and specific pathways of common four autoimmune diseases, including Type 1 Diabetes Mellitus (T1D), Multiple Sclerosis (MS), Rheumatoid Arthritis (RA) and Systemic Lupus Erythematosus (SLE). The results showed that Lysosome and Fc gamma R-mediated phagocytosis are shared pathways of the four diseases. It means that the occurrence and development of them may associate with lysosomes and phagocytosis. And there were 2 pathways are shared pathways of three diseases*,* ribosome pathway associated with susceptibility to MS, RA and SLE, and Pathogenic *Escherichia coli* infection associated with susceptibility to T1D, MS and RA; 9 pathways are shared pathways of two diseases. The corporate underlying causes of these diseases may be these shared pathways activated. Furthermore, we found that T1D-related specific pathways (Insulin signaling,etc.) were 9, MS (Proteasome,etc.) is also 9, RA and SLE is 10 and 6 respectively. These pathways could help us to reveal shared and specific mechanisms of the four diseases.

## INTRODUCTION

The frequent occurrence of human autoimmune diseases has effect on 5% of worldwide population, and it’s an increasing burden of morbidity and mortality on human beings. Autoimmune diseases are defined as diseases which an immune response occurred in the host body against autologous tissues and a pathological state of the host tissue damage as to lack of self-tolerance. Autoimmune diseases may arise in certain organs or particular tissues in different places, where are targeted by tissue-specific antigens, or may also involve whole-body tissues simultaneously, which have effect on multiple tissues and are targeted by all kinds of ubiquitously expressed autoantigens. Most of them are more common in females, and involve offspring trending genetics. Generally, the course of disease is long and more protracted chronic, and the pathological mechanism is not clear [1].

The expressions of disease-related genes are changed by environments controlling to altering epigenetic regulatory mechanism of gene expression. The correlation between autoimmune diseases and gene expression is defined as the basis of pathological mechanism . For example, the level of Foxp3 gene expression in the autoimmune diseases patients is lower than the normal [2]. Researchers also found many epigenetics factors (e.g. DNA methylation, microRNA (miRNA) and so on) related with autoimmune diseases. Richardson etc. discovered that DNA methylation is the key to maintain T-cell function. The failure of maintaining methylation levels and patterns in mature T-cell may result in autoreactive and autoimmunity. Lots of newly discovered miRNA regulation involves in innate and adaptive immune responses, immune cell development, T-cell stability and function, and differential miRNA expression specifically [3].

Autoimmune diseases share several diseases mechanism as a result they may have same (or similar) characteristics or same (or similar) etiologies. Recently, Tim-4 was found as a dysregulation in many autoimmune diseases [4]. Hironori’s researches also found polymorphisms of CTLA4, as primary susceptibility loci CTLA4 impact on the expression level of alternative splice of negative regulatory elements in T lymphocyte cell immune response [5]. Autoimmune diseases may often occur as overlap that two or more appeared on the same patient [6]. Autoimmune diseases have a genetic predisposition to family that autoimmune diseases family members are more likely to suffer from autoimmune diseases than non-family members, and they may suffer from different types of autoimmune diseases. However, several different autoimmune diseases show the same phenotypic characteristics such as multiple sclerosis and antiphospholipid syndrome, which are defined as neurological disorders and have similar clinical manifestations. The phenomenon of “overlap” and “familial aggregation” adumbrates the necessary link among autoimmune diseases, while autoimmune diseases are also characterized by themselves with showing different clinical phenotypes. Therefore the research of generality and specificity among autoimmune diseases is considered as a breakthrough for studying the pathogenesis of autoimmune diseases.

Autoimmune diseases are not given rise by a single factor and they are caused by a series of inducements, molecules, cellular pathways, phenotypes and events. Autoimmune diseases result from a complex alternate effect of pathways and phenotypes, which initially allow to begin from autoreactivity to manifest, then an initiating event of autoreaction, and finally allow development of self-sustaining tissue damage [7]. Recent researches have identified several risk pathways associated with the four common autoimmune diseases, such as immune response pathways, signaling pathways, transcriptional regulatory pathways, metabolic pathways and so on [8]. P53/p21 pathway and interferon pathway have been affirmed associated with SLE . Genome-wide data research conducted by our team had found 9 risk pathways related with RA, including focal adhesion, extracellular matrix-receptor interaction, calcium signaling, dopaminergic synapse, long-term potentiation, retrograde endocannabinoid signaling, glutamatergic synapse, cholinergic synapse and morphine addiction [8]. Analysis of disease-related risk pathways may help for understanding the pathogenesis of these diseases.

Currently, more than 80 autoimmune diseases have effect on approximately 100 million people worldwide [9], and among these diseases T1D, MS, RA and SLE are more common and more investigative. In summary, the significance of this research is to study the shared and specific characteristics of the four diseases by analyzing the shared and specific pathways and discovering the shared and specific pathological mechanism based on systems biology, that through integrating data of genetic level (such as expression profiling, SNP) and epigenetic level (such as methylation, miRNA), to mine disease-related genes.

## RESULTS

### Four related-disease genes

We conducted different genes researches on three different types’ data (see method), and found significantly different genes in every research (Supplementary Table 2). Among the 70 groups of case-control data on different diseases, there was one group that the number of different genes is greater than 1500, 6 groups between 1000 and 1500, and the others less than 1000. We merged the significantly different genes of three different types of data of each disease as a disease-related gene set. That is, the different genes above results calculated union on each type of data of every disease which is defined as the candidate gene sets. Then, we merged with a collection on genes of different types’ data with each disease that defined as disease-related candidate gene sets. Finally, we compared four diseases candidate gene set with disease-related gene databases existed to supple genes, which defined as disease-related gene sets (Table 2). The results contained different genes of methylation, mRNA and miRNA and target genes mapping the three-disease-associated SNP database (RADB http://www.bioapp.org/RADB/, TiDbase https://www.t1dbase.org/page/Welcome/display, MSGene http://www.msgene.org/) and four diseases related to genes that are validated from GAD (http://geneticassociationdb.nih.gov/).

These four autoimmune diseases share a familial genetic tendency, while they have different clinical manifestations. In order to understand the shared genetic tendency and specific clinical phenotypes, in our research we found out expression and regulation genes related to these diseases, and analyzed the shared and specific disease-related pathways.

### Shared genes and GO terms

According to the four related-disease genes above, we calculated the intersection of the four sets and obtain 33 genes the four diseases shared (Table 3). For instance, MIF (macrophage migration inhibitory factor) is a macrophage migration inhibitory factor, also it is a glycosylated inhibitor, and it related with immunoreaction of immune system. Calandra etc. research found that MIF is an important part of the body’s antimicrobial alarm system and immune cell proinflammatory function stress [13]. MIF is the constituent component of constituting inflammatory and it is the pathogenesis of autoimmune diseases [13]. Also, TGFB1 (transforming growth factor, beta 1) is a polypeptide of cytokine transforming growth factor involved in cell growth, cell proliferation, cell differentiation and apoptosis. TGFB1 produces and controls immune cells by lymphocytes, macrophages and dendritic cells, and thus it becomes an important component on the pathogenesis of autoimmune diseases [14].In order to further understand the function of these genes and the relationship between them and the four diseases and also to explore the pathogenesis of the diseases, we used the online annotation tool DAVID (https://david.ncifcrf.gov/).

We conducted these genes analyzing GO functional annotation, 27 genes were annotated in GO’s biological process (BP) branch which showed they are enriched in the immune response, negative regulation of endocytosis, negative regulation of cellular component organization and so on, 26 genes were annotated in cell component (CC) branch these genes are related to cell soma, lysosomes and lytic vacuole, which indicates that cellular components involved in the development and progression of autoimmune diseases, and 23 genes in molecular function (MF) branch are related with peptidase activity, which suggests that the mechanism of pathology of autoimmune diseases may related to the activity of peptidases *in vivo* (Table 4).

### Mining shared pathways and GO terms of the four diseases

We used DAVID to annotate four related-disease genes on GO and KEGG. The results show 59 shared terms containing 33 in BP branch, 22 in CC branch and 4 in MF branch (Table 5), which involve RNA processing, phosphorus metabolic process, phosphate metabolic process, apoptosis, humoral immune response, cell cycle, regulation of protein kinase cascade, regulation of cell death, positive regulation of cell death, programmed cell death, phosphorylation, regulation of protein modification process, positive regulation of protein modification process, regulation of cellular protein metabolic process, negative regulation of cellular protein metabolic process, positive regulation of cellular protein metabolic process, ncRNA processing, regulation of phosphorylation, regulation of apoptosis, positive regulation of apoptosis, regulation of programmed cell death, positive regulation of programmed cell death, positive regulation of catalytic activity, positive regulation of molecular function, innate immune response, positive regulation of protein kinase activity, regulation of binding, regulation of DNA binding, positive regulation of protein metabolic process, negative regulation of protein metabolic process, positive regulation of transferase activity, regulation of cell cycle, macromolecular complex assembly, nucleotide binding, protein kinase activity, enzyme binding, protein dimerization activity, cell fraction, lytic vacuole, nucleoplasm, nucleolus, mitochondrion, mitochondrial envelope, lysosome, vacuole, cytosol, cytoplasmic membrane-bounded vesicle, cytoplasmic vesicle, organelle envelope, membrane-enclosed lumen, envelope, nuclear lumen, vesicle, membrane-bounded vesicle, melanosome, organelle lumen, mitochondrial part, pigment granule and intracellular organelle lumen. It suggests that abnormal DNA transcription, RNA translation and non-coding RNA regulation join in apoptosis, cell cycle and other biological processes or mitochondria, lysosomes and other cell components, and the body’s innate immune response and immune response that are caused by body’s metabolism changing that lead by abnormal cell and processes abnormal, and eventually cause disease.

**Figure 1:**
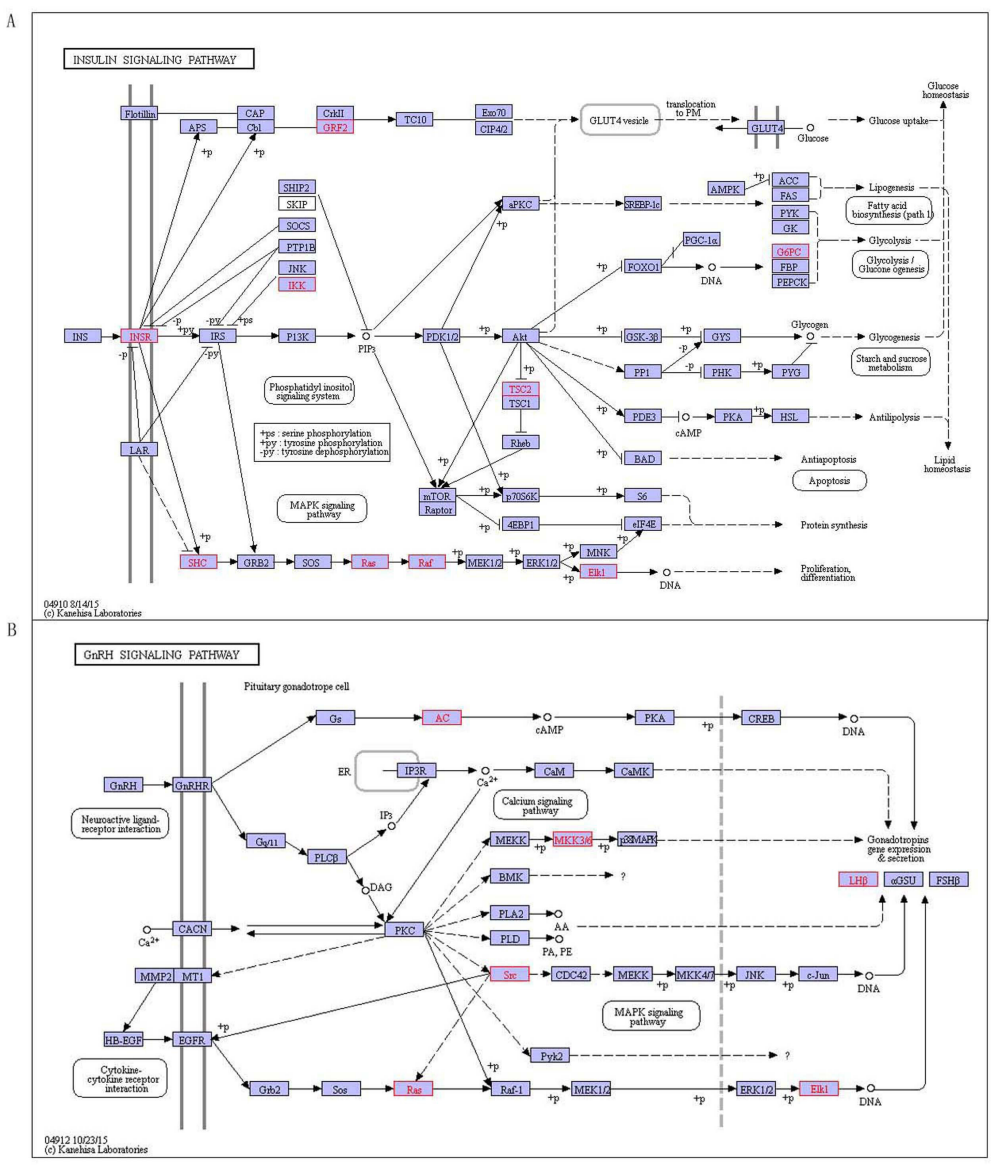
The shared pathways on four autoimmune diseases **A.** Lysosome pathway, **B.** Fc gamma R-mediated phagocytosis, red indicates disease-related genes.

We analyzed four disease pathways and discovery that there are 2 shared pathways associated with four diseases, 2 pathways are associated with three diseases, and nine pathways are associated with two diseases (Details in Table 6). Lysosome (hsa04142) and Fc gamma R-mediated phagocytosis (hsa04666) pathways are shared four diseases, details in Figure 1. They combine with each other in function and participate in the formation of lysosomes and phagocytosis. Lysosomal pathway is the interactional relationship between enzyme and genes described in cells. Lysosomes are membrane-delimited organelles in animal cells and serve as main digestive compartment in which all kinds of macromolecules are delivered and depredated. Lysosomes contain more than 40 hydrolases in an acidic environment (pH of about 5). Lysosomes synthesize in the endoplasmic reticulum (ER), and they are decorated with mannose-6-phosphate residues that are recognized by mannose-6-phosphate receptors in the trans-Golgi network [15]. They are packaged into clathrin-coated vesicles and then they are transported to late endosomes. Lysosomes acquire substances for digestion *via* a series of processes including endocytosis, phagocytosis, and autophagy . Phagocytosis joins in lysosomal pathway [15]. Fc gamma R-mediated phagocytosis pathway is a phagocytosis involving Fcγ receptor in intracellular. Phagocytosis composites the host-defense mechanisms that infectious pathogens absorb and destroy [16]. In higher organisms, these special cells including macrophages, neutrophils and monocytes involved in phagocytosis which constitutes the cellular immune barrier . Fc gamma receptors recognize foreign extracellular materials after opsonization with antibodies (IgG). Cross-linking of Fc gamma receptors initiates a variety of signals that is mediated by tyrosine phosphorylation of multiple proteins, which lead to the formation of phagosomes through the actin cytoskeleton rearrangements and membrane remodeling . Undergoing fusion with lysosomes, nascent phagosomes begin to become maturation. The acquisition of lysosomal proteases and release of reactive oxygen are crucial for digestion and degradation of engulfed materials in phagosomes . Phagocytosis conjunct with the lysosomes and ultimately exert their role, the two processes are complement and support each other mutually but indispensable, both of which acceleration and interaction with each other. In summary, they are associated with lysosomes: lysosomal pathway is a process lysosome degrades macromolecules in a cell by phagocytosis. While Fc gamma R-mediated phagocytosis pathway is that phagosomes formed Fc gamma receptor recognize antibody combine with lysosomes and activates; they are important ways to immune system playing. It implies that autoimmune diseases pathology is most likely the change to transcription and translation of genes associated with the lysosomes and Fcγ receptor in the body [16], and phagocytosis of immune system attack the body’s itself, which lead to tissues or systemic damage and eventually generate disease. Variation of involving genes has been increased by environmental factors, which caused the epigenetic regulation factor including non-coding RNA to change that caused transcription and translation of their regulatory target genes (autoimmune disease-related genes) to change, and then caused disease. We discovered that lysosomes are shared GO terms, which it shows lysosomes are important organelles involving the four kinds of autoimmune diseases, and the lysosome-associated genes involve din the development and progression of them.

**Figure 2:**
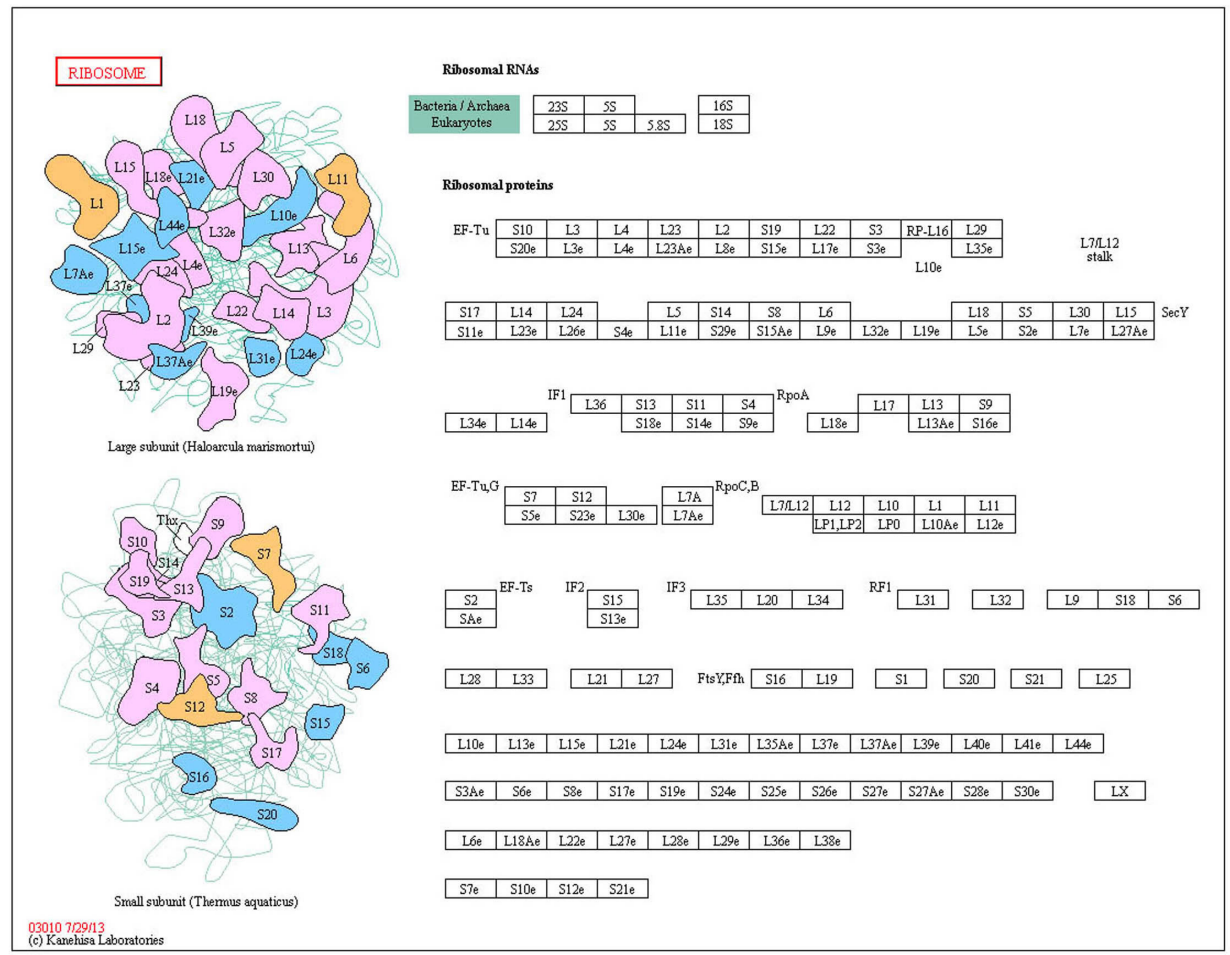
The shared ribosome pathway on MS RA and SLE, red indicates disease-related genes.

MS. RA and SLE shared the ribosome pathway (Figure 2). Ribosome is an essential organelle for translating and processing genetic information. It is a particle that composed of RNA and protein, and a lot of them exist in all active protein synthesis cells. Ribosome contains two subunits, formula weight of the large subunit is twice as the small, and their weights are about 60% of RNA. The small subunit determines the protein sequences constituting interactions mediated mRNA and tRNA, while the large catalyzes peptide bond formation. Aminoacyl tRNA (aa-tRNA) and peptidyl tRNA (p-tRNA) are substrate catalyzed large subunit, the former is bound to A-site in ribosome and the other combined P-site, α-amino group of AA-tRNA encountered 3 ‘hydroxyl carbon of carbonyl-acylated peptidyl -tRNA and formed on tetrahedral carbonyl carbon. Tetrahedral core is amino acids extending peptide, in which A-site is binding esterified tRNA and P binding deacylated tRNA, and then the new replacement of aa-tRNA and p-tRNA continue to extend [17]. The pathway involving in protein synthesis suggests that three diseases including MS, RA and SLE are related to ribosome synthesizing protein.

**Figure 3:**
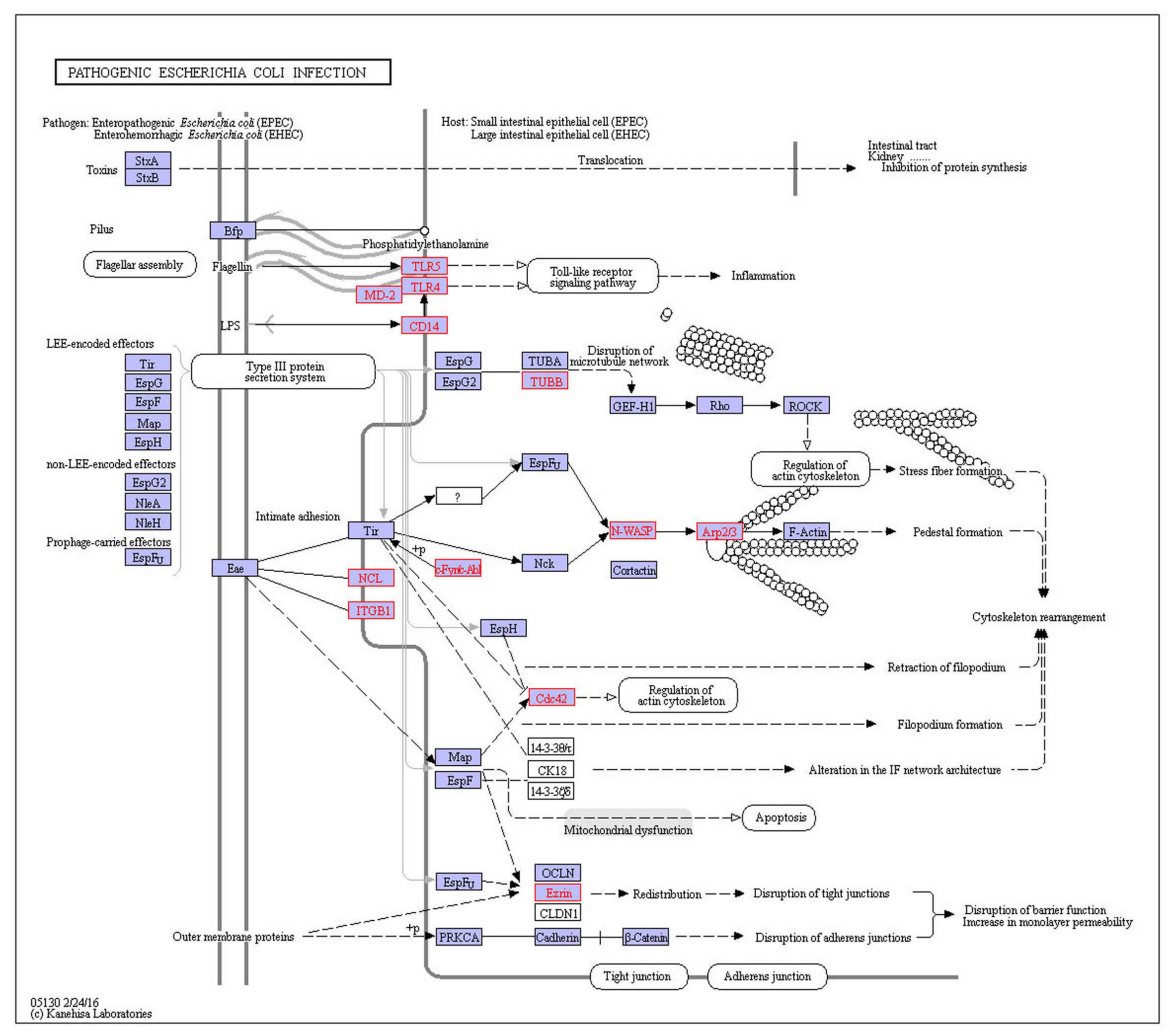
The shared pathogenic *Escherichia coli* infection pathway on T1D, MS and RA Red indicates disease-related genes.

T1D, MS and RA shared Pathogenic *Escherichia coli* infection pathway. Pathogenic *Escherichia coli* contain Enteropathogenic *E. coli* (EPEC) and enterohemorrhagic *E. coli* (EHEC) in pathway (Figure 3), they are closely related to pathogenic strains of *Escherichia coli*. The hallmark of EPEC/EHEC infections is an induction of damaging intestinal epithelial cells by attaching and effacing (A/E) lesions. The locus of enterocyte effacement (LEE) Pathogenicity Island encodes protein that is the capacity to form A/E lesions. Tir, Map, EspF and EspG are all LEE-encoded effector proteins secreted through the type III secretion system, which is also LEE-encoded into the host cell [18]. EPEC and EHEC Tir link the extracellular bacterium to the cell cytoskeleton, Map and EspF involve membrane permeabilization in mitochondrion [18]. EspG activates with tubulins and then stimulates microtubule destabilization. LEE-encoded adhesin or intimin (Eae) is exported through the general secretory pathway to the periplasm, where it is inserted into the outer membrane [19]. In addition to Tir, two potential host cell-carried intimin receptors, beta1 integrin (ITGB1) and nucleolin (NCL), have been identified so far. The remarkable feature of EHEC is the elaboration of Shiga-like toxin (Stx). Stx cleaves ribosomal RNA (rRNA), thereby disrupting protein synthesis and killing the intoxicated epithelial or endothelial cells. The pathway disrupting protein synthesis suggests that the development of three diseases including T1D, MS and RA is closely related to protein of pathway interference.

**Figure 4:**
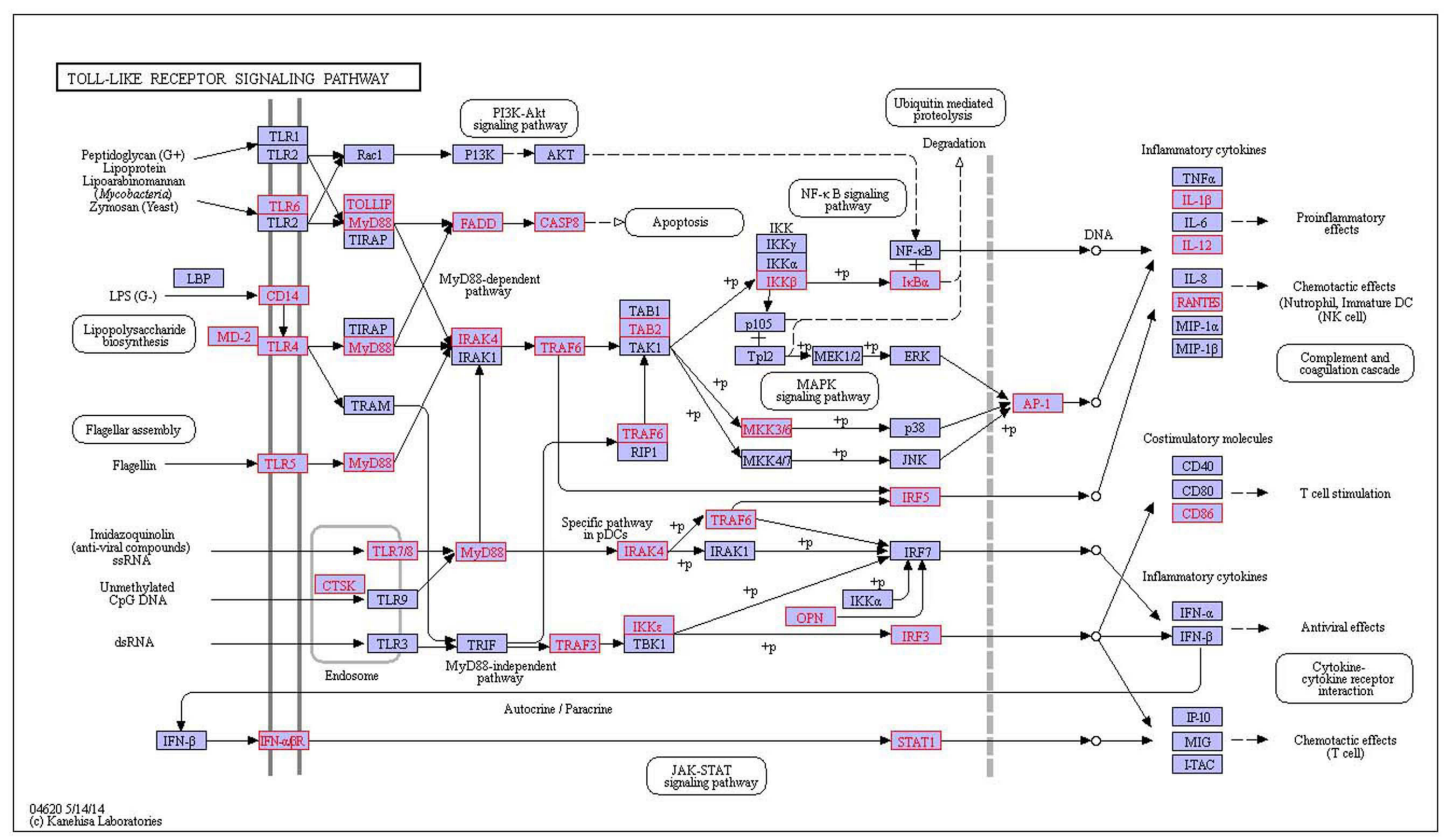
The shared toll-like receptor signaling pathway on RA and SLE, red indicates disease-related genes

The chronic myeloid leukemia pathway is shared by T1D and MS, the oxidative phosphorylation pathway is shared by MS and SLE, neurotrophin signaling pathway is shared by MS and RA, and chemokine signaling pathway, adherens junction pathway, Toll-like receptor signaling pathway, Natural killer cell mediated cytotoxicity pathway, T cell receptor signaling pathway and leukocyte transendothelial migration pathway are shared by RA and SLE. For example, toll-like receptor signaling pathway (Figure 4) is shared by RA and SLE, in which toll-like receptors (TLRs) is a specific pattern recognition receptor [20]. Specific families of pattern recognition receptors are used to detecting microbial pathogens and generating innate immune responses. Mammalian TLRs are expressed on innate immune cells including macrophages and dendritic cells, and reaction the membrane components of Gram-positive or Gram-negative bacteria [21]. TLRs recognition pathogen provokes rapidly activation of innate immunity based on inducing production of proinflammatory cytokines and upregulation of costimulatory molecules [21]. TLR signaling pathway is divided into two groups: a MyD88-dependent pathway that lead to be rapid activate NFκB and MAPK by the production of proinflammatory cytokines, and the other MyD88-independent pathway is associated with the induction of IFN-beta and IFN-inducible genes, and slow activation of NFκB and MAPK by maturation of dendritic cells [20]. The pathway has an effect on the activated process of NFκB and MAPK which is divided into two kinds of rapid and slow activation pathway, while RA and SLE is characterized by the acute onset and the long-term chronic cumulative which inducing tissue damage [22]. It indicates that the occurrence and development of RA and SLE are closely related with pathway and genes and cytokine in pathway.

The shared GO terms and KEGG pathways may provide a more valuable reference and basis for studying pathogenesis of autoimmune diseases.

### Mining specific pathways of the four autoimmune diseases

We discovered 34 specific pathways, for T1D is 9, MS is 9, RA is 10 and SLE is 6(detailed in Table 7-10). 19 of them have literature confirmation that they are related to the four diseases, in particular 9 specific pathways were found for T1D, among which 6 pathways have been confirmed related with SLE.

**Figure 5:**
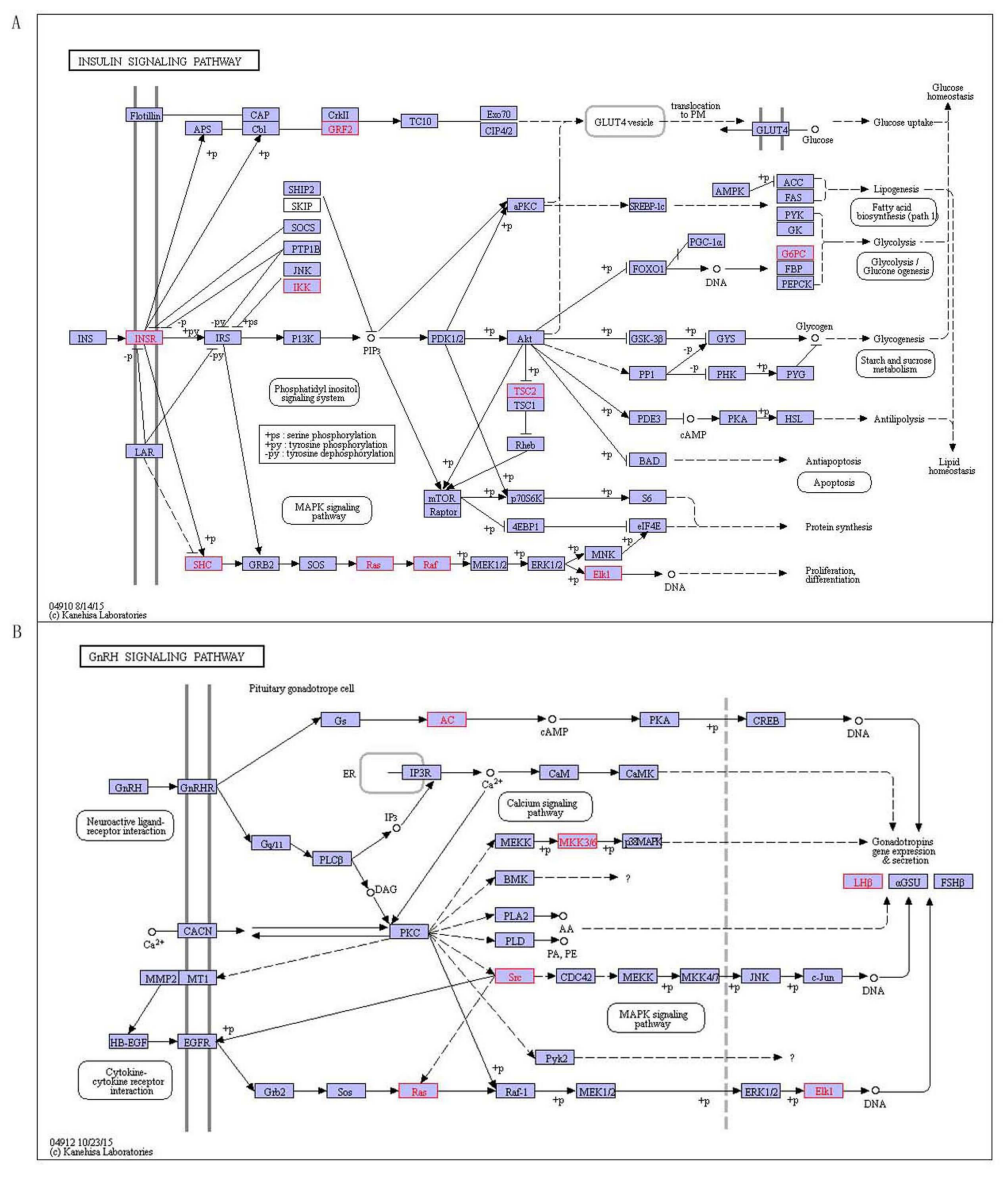
The special pathways of T1D, red indicates disease-related genes **A.** Insulin signaling pathway, **B.** GnRH signaling pathway.

There are 9 pathways related with T1D including histidine metabolism, glutathione metabolism, other glycan degradation, spliceosome, endocytosis, TGF-beta signaling pathway, insulin signaling pathway, GnRH signaling pathway and Vibrio cholerae infection(Table 7). Recent research has confirmed 6 pathways related with T1D, including histidine metabolism, glutathione metabolism, spliceosome, TGF-β signaling pathway, insulin signaling pathway and Vibrio cholerae infection. As an example to insulin signaling pathway (Figure 5A), which is binding insulin with receptor what makes insulin receptor substrate (IRS) occurring tyrosine phosphorylation by tyrosine kinase (INSR). This allows IRSs associated with the regulatory subunit of phosphoinositide 3-kinase (PI3K), and then PI3K activates 3-phosphoinositide-dependent protein kinase 1 (PDK1), which activates a serine kinase (Akt). In turn, Akt deactivates glycogen synthase kinase 3 (GSK-3), which leads to activation of glycogen synthase (GYS) and thus synthesizes glycogen. Activation of Akt also makes GLUT4 vesicles translocate from their intracellular pool to the plasma membrane, where glucose is allowed uptake into the cell. Akt also leads to activate the protein synthesis with mTOR-mediated by eIF4 and p70S6K [23]. The translocation of GLUT4 protein makes the CAP/Cbl/TC10 pathway elicite, once Cbl is phosphorylated by INSR. Other signal transduction proteins including GRB2 activate with IRS, which is part of the cascade including SOS, RAS, RAF and MEK that leads to mitogen-activated protein kinase (MAPK) activating and mitogenic responding in the form of gene transcription [23]. SHC is another substrate of INSR. When tyrosine phosphorylated, SHC may associate with GRB2 and thus activate the RAS/MAPK pathway of IRS-1 independently. The pathway increases in involving in glucose metabolism and controlling blood sugar levels in the body’s, and it is closely related with T1D. Insulin signaling pathway is the special characteristic of diabetes, diabetes is characterized by the body producing insulin abnormally, and diabetes patients also have a speed disorder of their body, which may be related to hormonal signal transduction pathway that leading to hormone levels abnormal. In addition, we predicted 3 pathways including other glycan degradation, endocytosis and GnRH signaling pathway related to T1D, wherein the GnRH signaling pathway (Figure 5B) is a process that Gonadotropin-releasing hormone (GnRH) secreting from the hypothalamus acts upon its receptor in the anterior pituitary and increases the production and releases the gonadotropins containing LH and FSH [24]. The GnRHR coupled to Gq/11 proteins activates phospholipase C which transmits its signal to diacylglycerol (DAG) and inositol 1, 4, 5-trisphosphate (IP3) [24]. DAG can activate the intracellular protein kinase C (PKC) pathway and IP3 can stimulate release of intracellular calcium. Occasionally, coupling of Gs except the classical Gq/11 is observed in a cell-specific fashion. Signaling downstream of PKC transactives the epidermal growth factor (EGF) receptor and actives mitogen-activated protein kinases (MAPKs), including extracellular-signal-regulated kinase (ERK), Jun N-terminal kinase (JNK) and p38 MAPK. Active MAPKs transfer into the nucleus, resulting in activating transcription factors and rapidly inducting early genes [25].

**Figure 6:**
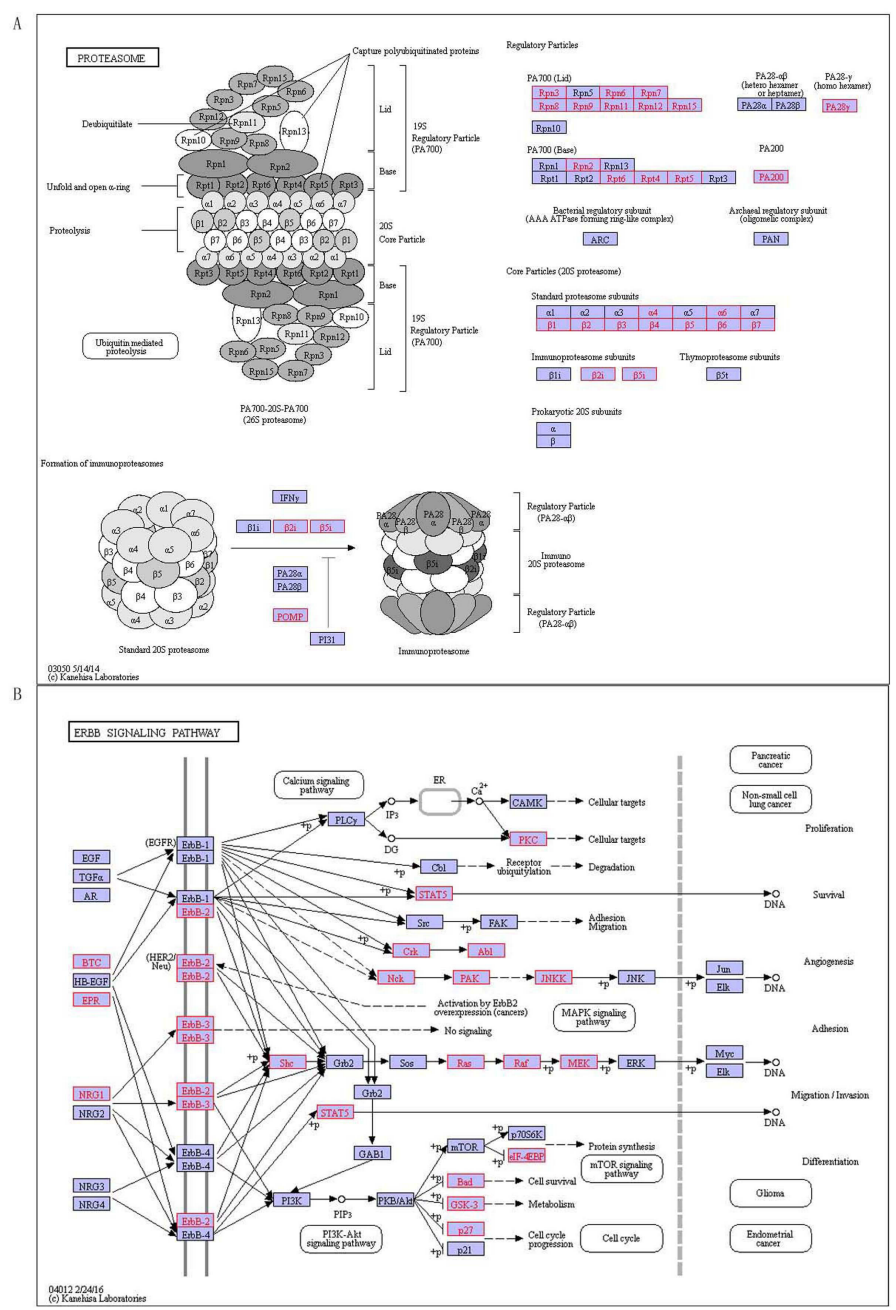
The special pathways of MS, red indicates disease-related genes **A.** Proteasome, **B.** ErbB signaling pathway.

There are 9 pathways related to MS, containing fructose and mannose metabolism, glycosaminoglycan degradation, proteasome, ErbB signaling pathway, mTOR signaling pathway, Alzheimer’s disease, Parkinson’s disease, Huntington’s disease and renal cell carcinoma (Table 8). Most of these pathways involve in the body’s nerves regulating and regulation [26], in which six are confirmed MS-related by literatures, including the proteasome, mTOR signaling pathway, Alzheimer’s disease, Parkinson’s disease, Huntington’s disease and renal cell carcinoma pathway. As an example, proteasome pathway is the main pathway that proteases activated in the body. Proteasome is a complex degrading protein in Figure 6A, which involves in many essential cellular functions, such as regulation of cell cycle, cell differentiation, apoptosis, signal transduction pathways, antigen processing for appropriate immune responses, stress signaling, and inflammatory responses . It is in a rapid and timely fashion that proteasome degrades a variety of cellular proteins and most substrate proteins are modified by ubiquitin before their degraded by the proteasome. PA200 has been identified as a large nuclear protein stimulating proteasomal hydrolysis of peptides [27]. The other three have not been confirmed associated with MS, there they are predicted for the first time, including fructose and mannose metabolism, glycosaminoglycan degradation and ErbB signaling pathway. ErbB signaling pathway stimulates diverse biologic responses that ErbB family of receptor tyrosine kinases (RTKs) couples binding extracellular growth factor ligands to intracellular signaling pathways (Figure 6B), which includes proliferation, differentiation, cell motility and survival. Ligand binding to the four closely related members of RTK family, epidermal growth factor receptor (EGFR, also known as ErbB-1 or HER1), ErbB-2 (HER2), ErbB-3 (HER3), and ErbB-4 (HER4), induces forming receptor homo- and heterodimers and actives intrinsic kinase domain, and then results in phosphorylation on specific tyrosine residues (pY) within the cytoplasmic tail . The Shc- and/or Grb2-activated mitogen-activated protein kinase (MAPK) pathway is a shared target downstream of all ErbB receptors [28]. Similarly, the phosphatidylinositol-3-kinase (PI-3K) pathway is directly or indirectly activated by most of ErbBs [28]. Several cytoplasmic docking proteins seem to be exploited by specific ErbB receptors and less by others . We found that these specific pathways of our research are involved in an important factor of MS development. More interesting, there are three pathways containing Alzheimer’s disease, Parkinson’s disease, and Huntington’s disease are involved in MS and other three neurological diseases. The results imply that MS is closely related with Alzheimer’s disease, Parkinson’s disease and Huntington’s disease.

**Figure 7:**
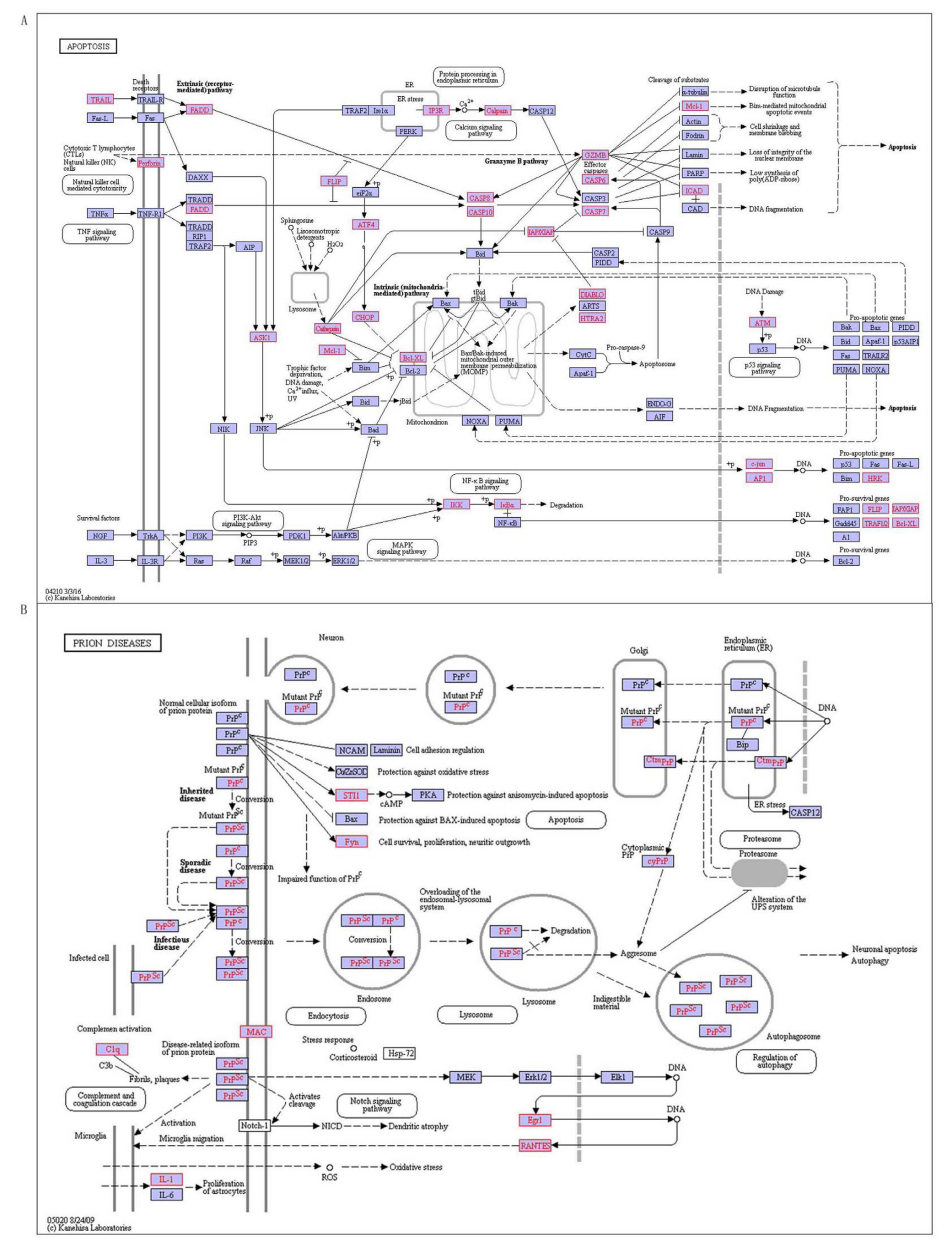
The special pathways on RA, red indicates disease-related genes **A.** Apoptosis, **B.** Prion diseases.

There are 10 pathways associated with RA (Table 9), containing cysteine and methionine metabolism, cytokine-cytokine receptor interaction, apoptosis, hematopoietic cell lineage, B cell receptor signaling pathway, Fc epsilon RI signaling pathway, and intestinal immune network for IgA production, circadian rhythm, prion diseases and primary immunodeficiency. Previous researches have demonstrated four pathways associated with RA including apoptosis, B cell receptor signaling pathways, circadian rhythm and primary immunodeficiency. Apoptosis pathway is a genetically programmed process to eliminate damaged or redundant cells by caspases (aspartate-specific cysteine proteases) activing (Figure 7A). The occurrence of apoptosis is controlled by numerous interrelating processes [29]. The ‘extrinsic’ pathway involves in stimulating members of tumor necrosis factor (TNF) receptor subfamily, such as TNFRI, CD95/Fas or TRAILR (death receptors) which locate on the cell surface, by their specific ligands such as TNF-alpha, FasL or TRAIL, respectively [29]. The ‘intrinsic’ pathway is activated mainly by non-receptor stimuli that containing DNA damage, ER stress, metabolic stress, UV radiation or growth-factor deprivation [29]. The central event of the ‘intrinsic’ pathway is the mitochondrial outer membrane permeabilization (MOMP), and then leads to the release of cytochrome C. These two pathways converge at the level of effector caspases for instance caspase-3 and caspase-7. The constituents of cytotoxic (e.g. Perforin and Granzyme B) granules initiates the third major pathways that are released by CTLs (cytotoxic T-cells) and NK (natural killer) cells. Granzyme B is similar to the caspases cleaves its substrates after aspartic acid residues, which suggests that this protease has been able to activate members of the caspase family directly. The balance between the pro- and anti-apoptotic signals eventually determines whether cells will undergo apoptosis, survive or proliferate. We predicted 6 pathways related with RA, including cysteine and methionine metabolism, cytokine-cytokine receptor interaction, hematopoietic cell lineage, Fc epsilon RI signaling pathway, intestinal immune network for IgA production, and Prion diseases. We found that one of RA-related pathways - prion diseases pathway, is a caused process of body reaction by prions in Figure 7B. Prion diseases, also called transmissible spongiform encephalopathies (TSEs), are a series of fatal neurodegenerative diseases that have an effect on humans and a number of other animal species. The etiology of them is considered to be associated with converting a normal protein PrPC into an infectious pathogenic form PrPSc. The conversion is induced by prion infections (for example, variant Creutzfeldt-Jakob disease (vCJD), iatrogenic CJD, Kuru), mutations (familial CJD, Gerstmann-Straussler-Scheinker syndrome, fatal familial insomnia (FFI)) or unknown factors (sporadic CJD (sCJD)), and it is considered to occur after PrPC has reached the plasma membrane or has been re-internalized for degradation [30]. The PrPSc form shows greater protease resistance than PrPC, which accumulates in affected individuals and often in the form of extracellular plaques [31]. Pathways lead to neuronal death comprise oxidative stress, corticosteroid response, endoplasmic reticulum stress, regulated activation of complement, ubiquitin-proteasome and endosomal-lysosomal systems, synaptic alterations and dendritic atrophy [31]. In addition, the conformational transition could lead to loss of a beneficial activity that protein PrPC is the natively folded [31]. It is more interesting that the specific pathways of RA are related with circadian rhythm, which suggests that the occurrence of RA is closely related to environmental factors. The findings provide a better and more effective ways to prevent the development and progression of RA.

**Figure 8:**
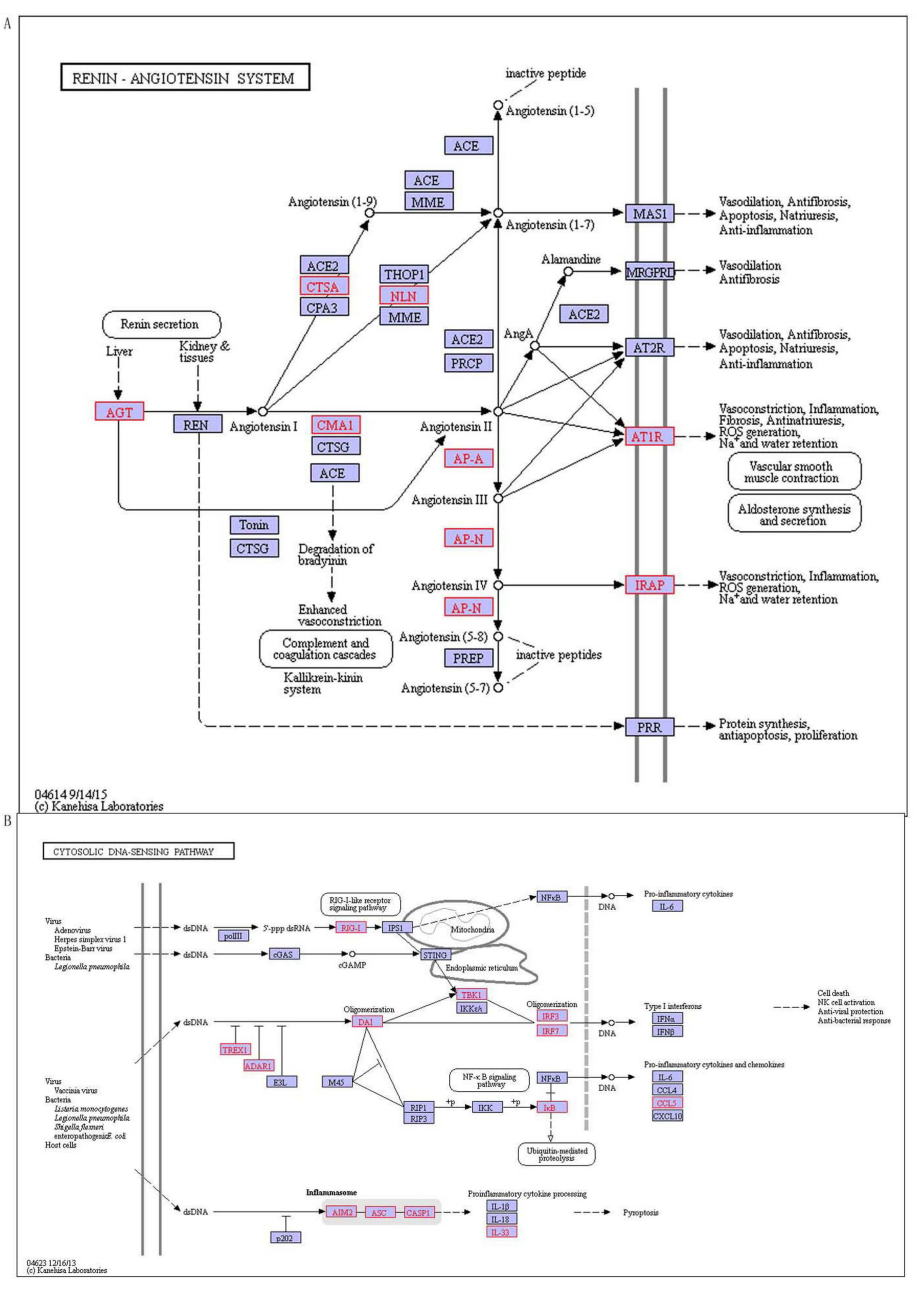
The special pathways of SLE, red indicates disease-related genes **A.** Renin-angiotensin system, **B.** Cytosolic DNA-sensing pathway.

There are 6 SLE specific pathways (Table 10), containing MAPK signaling pathway, calcium signaling pathway, renin-angiotensin system, RIG-I-like receptor signaling pathway, cytosolic DNA-sensing pathway and prostate cancer, they are signal pathways involved in regulating systemic and systemic vascular regulation, and these features are coincided with characteristics occurring SLE systemic. There are 3 of them confirmed they are related to SLE by literatures, including calcium signaling pathway, renin-angiotensin system and prostate cancer. For example renin-angiotensin system (RAS) is a peptidergic system with endocrine regulation of the blood pressure and hydro-electrolytic balance (Figure 8A). In the classical RAS, the enzyme renin cleaves its substrate angiotensinogen (Agt), and it is forming the decapeptide angiotensin I that is cleaved by angiotensin-converting enzyme (ACE) and produces the angiotensin II (Ang II) in turn, which is a key player of this system [32]. Ang II activates its AT1 receptor (AT1R), the mainly receptor mediates the majority of Ang II as known actions in the kidney including vasoconstriction, renal sodium (Na+) reabsorption, and aldosterone secretion, increasing blood pressure and contributing to the development of hypertension. In addition to (ACE)/Ang II/AT1R and AT2R axis, other signaling pathways in the RAS( such as ACE2/angiotensin-(1-7)/Mas and Ang IV/IRAP), and other active peptide of the RAS(physiological relevance as Ang III, Ang A and alamandine), are now also widely recognized [33]. RAS is a pathway with systemic impact, which activates the body’s water and electrolyte regulation. This means that the pathogenesis of SLE is associated with RAS. Furthermore, we also firstly observed MAPK signaling pathway, RIG-I-like receptor signaling pathway and cytosolic DNA-sensing pathway with SLE-related. Among them, cytosolic DNA-sensing pathway has an effect on systemic pathway (Figure 8B), and it is innate immune responses generated that specific families of pattern recognition receptors are responsible for detecting foreign DNA from invading microbes or host cells . DAI is the first sensor identified cytosolic DNA which activates the IRF and NF κ B transcription factors and leads to production of type I interferon and other cytokines. AIM2 is also a type of cytoplasmic DNA sensor. Upon sensing DNA, AIM2 triggers off assembling inflammasome, and culminates in interleukin maturation. In addition, to these receptors there is a mechanism to sense foreign DNA with the host RNA polymerase III converting the DNA into RNA recognized by the RNA sensor RIG-I [34]. These pathways alert the cell through various means, and are closely related with SLE.

In brief, the specific pathways provide theoretical basis and reference of understanding specific phenotypes and mechanisms.

## DISCUSSION

Our research explored the shared and special mechanisms of four common autoimmune diseases including T1D, MS, RA and SLE. Their occurrences are result from abnormal immune system that antibodies attacks normal tissues lead to damaged tissues and organs, these four diseases acquire some shared features that maybe also shared some features of autoimmune diseases. The shared features may be controlled by their shared genes and pathways. They also have their own specific related genes and pathways. When they occur as abnormalities, those diseases will appear disease-specific clinical phenotype.

This research used four different types function data (mRNA expression profile, SNP, methylation, microRNA expression profile), to study the shared and specific mechanisms of four autoimmune diseases from genetics and epigenetics. The purpose of this research is studying how miRNA and methylation regulate its target SNP and mRNA expression by the four different levels data, which affects the body’s normal pathways access connectivity change and produce disease. We discover that there are 2 shared pathways associated with four diseases, including Lysosome (hsa04142) and Fc gamma R-mediated phagocytosis (hsa04666), and they are closely linked with each other. When lysosomes is formatting, the phagocytosis plays a necessary role, and contrarily phagocytosis results in forming lysosomes. That is illustrated that the occurrence and development of these four autoimmune diseases may be associated with lysosomes and phagocytosis. Since the genes that are controlling both pathways is influenced by genetic and environmental factors, cell lysosomes and phagocytosis have emerged abnormal in immune-related reaction, and then brought autoimmune diseases. At the same time, we also found 33 shared four disease-related genes, which are respectively annotated on GO and KEGG and found most of them are involving in immune system adjustment and response. It suggests that the change of expression and regulation cause transcription and translation, which is the important reason for causing the autoimmune diseases. The result makes pathogenesis and common mechanisms of autoimmune diseases take a forward step. Also, we found that T1D-related specific pathways were 9, MS is also 9, RA and SLE is 10 and 6 respectively. These pathways represent specific characteristics of their related to diseases, and so reveal specific mechanisms of the four diseases.

In our study, we carried out different test to genes or probes on the relation between probes and corresponding genes. In order to reduce the false positive rate and to improve the accuracy of the experiment as much as possible, we conducted a rigorous BH corrected and selected higher threshold, through which the genes are related to disease. While sample size is small , *T*-test is not siuitable, we can use fold-change to measure the differences and also adopt high standards as above for screening differences resulting in unified the number of different genes with each study. We used a larger number of samples, which is more widespread phenomenon, and then those special studies impact is not particularly serious on the overall. Our research methods ensure the results robust.

## MATERIALS AND METHODS

### Data

The expression, methylation and miRNA expression data used in this research were download from GEO database, which were acquired by searching keywords of the four diseases [Rheumatoid Arthritis, Multiple Sclerosis, Systemic Lupus Erythematosus, Diabetes Mellitus Type 1] and then the original data were filtered: the case-control data of T1D have 10 groups including one methylation, and 9 mRNA expression profiling of six different platforms, details in Supplementary Table 1; the case-control data of MS have 23 groups including one methylation, 16 mRNA expression profiling of 9 different platforms, and 6 miRNA expression profiling of 5 platforms, details in Supplementary Table 1; the case-control data of RA have 23 groups including two methylation of two platforms, 17 mRNA expression profiling of 14 different platforms, 5 miRNA expression profiling of 5 platforms, details in Supplementary Table 1; the case-control data of SLE have 18 groups including two methylation of two platforms, 14 mRNA expression profiling of 9 different platforms, 2 miRNA expression profiling of 2 platforms, details in Supplementary Table 1. The case-control sample data used in this research totally contain 70 groups of 36 different platforms, and the data statistics of the three different types on these four diseases are shown in Table 1. Among these data, the data size of MS and RA are larger than the others, and expression profiles is the most studied in the three types of data.

**Table 1:** The quantity of researches on the three types’ data (methylation, expression and miRNA) related to the four diseases

Disease	Methylation	Expression	miRNA
T1D	1	9	0
MS	1	16	6
RA	2	16	5
SLE	2	14	2

RA : Rheumatoid Arthritics, MS : Multiple Sclerosis, SLE : Systemic Lupus Erythematosus, T1D : Type: Diabetes, miRNA: microRNA

**Table 2:** The number of different genes of four diseases

Disease	T1D	MS	RA	SLE
Genes	1411	4946	4613	4201

**Table 3:** The shared genes related to the four diseases

GENE_SYMBOL	GENE_NAME	SPECIES
ARPC5L	actin related protein 2/3 complex, subunit 5-like	Homo sapiens
B2M	beta-2-microglobulin	Homo sapiens
C20orf24	chromosome 20 open reading frame 24	Homo sapiens
CCDC127	coiled-coil domain containing 127	Homo sapiens
CDC25B	cell division cycle 25 homolog B (S. pombe)	Homo sapiens
CLEC10A	C-type lectin domain family 10, member A	Homo sapiens
CLPP	ClpP caseinolytic peptidase, ATP-dependent, proteolytic subunit homolog (*E. coli*)	Homo sapiens
CTSZ	cathepsin Z	Homo sapiens
CYBA	cytochrome b-245, alpha polypeptide	Homo sapiens
DNAJB11	DnaJ (Hsp40) homolog, subfamily B, member 11	Homo sapiens
EEF1G	eukaryotic translation elongation factor 1 gamma	Homo sapiens
EPHB2	EPH receptor B2	Homo sapiens
FEM1C	fem-1 homolog c (C. elegans)	Homo sapiens
GGH	gamma-glutamyl hydrolase (conjugase, folylpolygammaglutamyl hydrolase)	Homo sapiens
KIAA1967	KIAA1967	Homo sapiens
MAN2B1	mannosidase, alpha, class 2B, member 1	Homo sapiens
MARCH1	membrane-associated ring finger (C3HC4) 1	Homo sapiens
MICU1	mitochondrial calcium uptake 1	Homo sapiens
MIF	macrophage migration inhibitory factor (glycosylation-inhibiting factor)	Homo sapiens
MRAS	muscle RAS oncogene homolog	Homo sapiens
NR1H3	nuclear receptor subfamily 1, group H, member 3	Homo sapiens
NXT1	NTF2-like export factor 1	Homo sapiens
PDCD7	programmed cell death 7	Homo sapiens
PGAM1	phosphoglycerate mutase 1 (brain)	Homo sapiens
PPDPF	pancreatic progenitor cell differentiation and proliferation factor	Homo sapiens
PREPL	prolyl endopeptidase-like	Homo sapiens
RNASET2	ribonuclease T2	Homo sapiens
RWDD1	RWD domain containing 1-like 1; RWD domain containing 1	Homo sapiens
SDF2L1	stromal cell-derived factor 2-like 1	Homo sapiens
SLC43A1	solute carrier family 43, member 1	Homo sapiens
TGFB1	transforming growth factor, beta 1	Homo sapiens
TREM2	triggering receptor expressed on myeloid cells 2	Homo sapiens
ZP3	zona pellucida glycoprotein 3 (sperm receptor)	Homo sapiens

**Table 4:** The shared genes annotating GO terms

Category	Term	*P* Value
GOTERM_BP_FAT	GO:0006955∼immune response	0.009161
GOTERM_BP_FAT	GO:0045730∼respiratory burst	0.02471
GOTERM_BP_FAT	GO:0045806∼negative regulation of endocytosis	0.028459
GOTERM_BP_FAT	GO:0045087∼innate immune response	0.028615
GOTERM_BP_FAT	GO:0051129∼negative regulation of cellular component organization	0.030163
GOTERM_BP_FAT	GO:0006611∼protein export from nucleus	0.034057
GOTERM_BP_FAT	GO:0048610∼reproductive cellular process	0.038386
GOTERM_BP_FAT	GO:0009057∼macromolecule catabolic process	0.059937
GOTERM_BP_FAT	GO:0045596∼negative regulation of cell differentiation	0.064192
GOTERM_BP_FAT	GO:0050768∼negative regulation of neurogenesis	0.083059
GOTERM_BP_FAT	GO:0045834∼positive regulation of lipid metabolic process	0.084827
GOTERM_BP_FAT	GO:0010721∼negative regulation of cell development	0.088353
GOTERM_BP_FAT	GO:0002706∼regulation of lymphocyte mediated immunity	0.098855
GOTERM_CC_FAT	GO:0043025∼cell soma	0.042278
GOTERM_CC_FAT	GO:0005764∼lysosome	0.063472
GOTERM_CC_FAT	GO:0000323∼lytic vacuole	0.063472
GOTERM_CC_FAT	GO:0005773∼vacuole	0.086351
GOTERM_MF_FAT	GO:0070011∼peptidase activity, acting on L-amino acid peptides	0.056796
GOTERM_MF_FAT	GO:0008233∼peptidase activity	0.063278

There are 13, 4 and 2 terms in BP, CC and MF respectively, which the number of genes is greater than 2. 7 of 13 BP’s terms and 1 of 4 CC’s terms are significantly enrichment.

**Table 5:** The shared genes in GO terms

Category	GO_TermID	GO_annotation
GOTERM_BP	GO:0006396	RNA processing
GOTERM_BP	GO:0006793	phosphorus metabolic process
GOTERM_BP	GO:0006796	phosphate metabolic process
GOTERM_BP	GO:0006915	apoptosis
GOTERM_BP	GO:0006959	humoral immune response
GOTERM_BP	GO:0007049	cell cycle
GOTERM_BP	GO:0010627	regulation of protein kinase cascade
GOTERM_BP	GO:0010941	regulation of cell death
GOTERM_BP	GO:0010942	positive regulation of cell death
GOTERM_BP	GO:0012501	programmed cell death
GOTERM_BP	GO:0016310	phosphorylation
GOTERM_BP	GO:0031399	regulation of protein modification process
GOTERM_BP	GO:0031401	positive regulation of protein modification process
GOTERM_BP	GO:0032268	regulation of cellular protein metabolic process
GOTERM_BP	GO:0032269	negative regulation of cellular protein metabolic process
GOTERM_BP	GO:0032270	positive regulation of cellular protein metabolic process
GOTERM_BP	GO:0034470	ncRNA processing
GOTERM_BP	GO:0042325	regulation of phosphorylation
GOTERM_BP	GO:0042981	regulation of apoptosis
GOTERM_BP	GO:0043065	positive regulation of apoptosis
GOTERM_BP	GO:0043067	regulation of programmed cell death
GOTERM_BP	GO:0043068	positive regulation of programmed cell death
GOTERM_BP	GO:0043085	positive regulation of catalytic activity
GOTERM_BP	GO:0044093	positive regulation of molecular function
GOTERM_BP	GO:0045087	innate immune response
GOTERM_BP	GO:0045860	positive regulation of protein kinase activity
GOTERM_BP	GO:0051098	regulation of binding
GOTERM_BP	GO:0051101	regulation of DNA binding
GOTERM_BP	GO:0051247	positive regulation of protein metabolic process
GOTERM_BP	GO:0051248	negative regulation of protein metabolic process
GOTERM_BP	GO:0051347	positive regulation of transferase activity
GOTERM_BP	GO:0051726	regulation of cell cycle
GOTERM_CC	GO:0065003	macromolecular complex assembly
GOTERM_CC	GO:0000166	nucleotide binding
GOTERM_CC	GO:0004672	protein kinase activity
GOTERM_CC	GO:0019899	enzyme binding
GOTERM_CC	GO:0046983	protein dimerization activity
GOTERM_CC	GO:0000267	cell fraction
GOTERM_CC	GO:0000323	lytic vacuole
GOTERM_CC	GO:0005654	nucleoplasm
GOTERM_CC	GO:0005730	nucleolus
GOTERM_CC	GO:0005739	mitochondrion
GOTERM_CC	GO:0005740	mitochondrial envelope
GOTERM_CC	GO:0005764	lysosome
GOTERM_CC	GO:0005773	vacuole
GOTERM_CC	GO:0005829	cytosol
GOTERM_CC	GO:0016023	cytoplasmic membrane-bounded vesicle
GOTERM_CC	GO:0031410	cytoplasmic vesicle
GOTERM_CC	GO:0031967	organelle envelope
GOTERM_CC	GO:0031974	membrane-enclosed lumen
GOTERM_CC	GO:0031975	envelope
GOTERM_CC	GO:0031981	nuclear lumen
GOTERM_CC	GO:0031982	vesicle
GOTERM_CC	GO:0031988	membrane-bounded vesicle
GOTERM_CC	GO:0042470	melanosome
GOTERM_MF	GO:0043233	organelle lumen
GOTERM_MF	GO:0044429	mitochondrial part
GOTERM_MF	GO:0048770	pigment granule
GOTERM_MF	GO:0070013	intracellular organelle lumen

**Table 6:** The four diseases-related pathways

Disease	T1D	MS	RA	SLE
pathway	hsa04142:Lysosome	hsa04142:Lysosome	hsa04142:Lysosome	hsa04142:Lysosome
	hsa04666:Fc gamma R-mediated phagocytosis	hsa04666:Fc gamma R-mediated phagocytosis	hsa04666:Fc gamma R-mediated phagocytosis	hsa04666:Fc gamma R-mediated phagocytosis
		hsa03010:Ribosome	hsa03010:Ribosome	hsa03010:Ribosome
	hsa05130:Pathogenic *Escherichia coli* infection	hsa05130:Pathogenic *Escherichia coli* infection	hsa05130:Pathogenic *Escherichia coli* infection	
	hsa05220:Chronic myeloid leukemia	hsa05220:Chronic myeloid leukemia		
		hsa00190:Oxidative phosphorylation		hsa00190:Oxidative phosphorylation
			hsa04062:Chemokine signaling pathway	hsa04062:Chemokine signaling pathway
			hsa04520:Adherens junction	hsa04520:Adherens junction
			hsa04620:Toll-like receptor signaling pathway	hsa04620:Toll-like receptor signaling pathway
			hsa04650:Natural killer cell mediated cytotoxicity	hsa04650:Natural killer cell mediated cytotoxicity
			hsa04660:T cell receptor signaling pathway	hsa04660:T cell receptor signaling pathway
			hsa04670:Leukocyte transendothelial migration	hsa04670:Leukocyte transendothelial migration
		hsa04722:Neurotrophin signaling pathway	hsa04722:Neurotrophin signaling pathway	

**Table 7:** The special pathways on T1D

Disease	ID	KEGG_pathway	Certified
T1D	1	hsa00340:Histidine metabolism	*
T1D	2	hsa00480:Glutathione metabolism	*
T1D	3	hsa00511:Other glycan degradation	
T1D	4	hsa03040:Spliceosome	*
T1D	5	hsa04144:Endocytosis	
T1D	6	hsa04350:TGF-beta signaling pathway	*
T1D	7	hsa04910:Insulin signaling pathway	*
T1D	8	hsa04912:GnRH signaling pathway	
T1D	9	hsa05110:Vibrio cholerae infection	*

*: The pathways that are certified associated with T1D by papers

**Table 8:** The special pathways of MS

Disease	ID	KEGG_pathway	Certified
MS	1	hsa00051:Fructose and mannose metabolism	
MS	2	hsa00531:Glycosaminoglycan degradation	
MS	3	hsa03050:Proteasome	*
MS	4	hsa04012: ErbB signaling pathway	
MS	5	hsa04150:mTOR signaling pathway	*
MS	6	hsa05010:Alzheimer's disease	*
MS	7	hsa05012:Parkinson's disease	*
MS	8	hsa05016:Huntington's disease	*
MS	9	hsa05211:Renal cell carcinoma	*

*: The pathways that are certified associated with MS by papers

**Table 9:** The special pathways of RA

Disease	ID	KEGG_pathway	Certified
RA	1	hsa00270:Cysteine and methionine metabolism	
RA	2	hsa04060:Cytokine-cytokine receptor interaction	
RA	3	hsa04210:Apoptosis	*
RA	4	hsa04640:Hematopoietic cell lineage	
RA	5	hsa04662:B cell receptor signaling pathway	*
RA	6	hsa04664:Fc epsilon RI signaling pathway	
RA	7	hsa04672:Intestinal immune network for IgA production	
RA	8	hsa04710:Circadian rhythm	*
RA	9	hsa05020:Prion diseases	
RA	10	hsa05340:Primary immunodeficiency	*

*: The pathways that are certified associated with RA by papers

**Table 10:** The special pathways of SLE

Disease	ID	KEGG_pathway	Certified
SLE	1	hsa04010:MAPK signaling pathway	
SLE	2	hsa04020:Calcium signaling pathway	*
SLE	3	hsa04614:Renin-angiotensin system	*
SLE	4	hsa04622:RIG-I-like receptor signaling pathway	
SLE	5	hsa04623:Cytosolic DNA-sensing pathway	
SLE	6	hsa05215:Prostate cancer	*

*: The pathways that are certified associated with SLE by papers

### Data procession and different genes filterion

Different types’ data means different: mRNA expression profiling data is a direct reflection on expression of the gene itself, methylation regulating target genes methylation level near the location of methylated sites can reflect gene expression, while miRNA regulating RNA sequences can inhibit genes expression.

(1) Filterion of different genes based on methylation and gene expression profile

Firstly, we can map probes into genes according to annotated platform file and when a gene contains a plurality of probes, we calculated the average of probes to measure the expression level or methylation level of the gene. Then, we can analyze every case-control research of every disease to filter out significantly different genes. Here, for the case-control samples with size greater than 2 *T*-test and Benjamini-Hochberg (BH) correction (*q* < 0.001) were used [10], and for the rest fold change method (FC > 2) was used [11].

(2) Filterion of different miRNA and target genes based on miRNA

We analyzed each case-control data of miRNA and filtered four diseases related to the different miRNA by using *T*-test and BH correction (*q* < 0.001) to the samples with size greater than 2 [10],and the rest through using fold change (FC > 2) to filter out the significant difference of miRNA [11]. Then, we determined target genes that expressed different miRNA based on the regulatory relationship between miRNA and target genes in miRTarBase (http://mirtarbase.mbc.nctu.edu.tw/). Because the same gene may be inhibited by many miRNAs, the standard of target genes filtered out is the miRNAs regulating target genes are all significant different miRNA and then the gene is different related-disease gene.

We got four related-disease gene sets, which are used following to analyze.

### Annotating GO and mining KEGG pathway based on the genes related to the four autoimmune diseases

We used online annotation tool DAVID for disease-related genes annotating of GO and KEGG [12]. We first analyzed and annotated the four related-disease genes, and discovered the shared genes, GO terms and KEGG pathways. Then we analyzed and discovered the specific GO terms and KEGG pathways related to the four disease genes.

## SUPPLEMENTARY MATERIALS TABLES





## Abbreviations

T1D: Type 1 Diabetes Mellitus
MS: Multiple Sclerosis
RA: Rheumatoid Arthritis
SLE: Systemic Lupus Erythematosus
SNP: Single Nucleotide Polymorphisms
miRNA: microRNA
