# Supplementary material for: The shared and specific mechanism of four autoimmune diseases
Source: Oncotarget. 2017 Jul 19;8(65):108355–74. doi: 10.18632/oncotarget.19383 (PMC5752449; doi:10.18632/oncotarget.19383)
Supplement: Supplementary file 1 [file oncotarget-08-108355-s001.pdf]

The shared and specific mechanism of four autoimmune diseases

SUPPLEMENTARY MATERIALS

For Supplementray Tables see in Supplementary Files
