# Supplementary material for: The shared and specific mechanism of four autoimmune diseases
Source: Oncotarget. 2017 Jul 19;8(65):108355–74. doi: 10.18632/oncotarget.19383 (PMC5752449; doi:10.18632/oncotarget.19383)
Supplement: Supplementary file 2 [file oncotarget-08-108355-s002.doc]

**Supplementary Table 1:** Case-control data of four diseases and information of platform

| Disease | ID | GSE | Case_number | Control_number | GPL | Type |
| --- | --- | --- | --- | --- | --- | --- |
| T1D | 1 | GSE56606 | 32 | 68 | GPL8490 | Mehtylation |
| T1D | 2 | GSE24147 | 21 | 21 | GPL570 | Expression |
| T1D | 3 | GSE29142 | 9 | 20 | GPL13507 | Expression |
| T1D | 4 | GSE30209 | 29 | 29 | GPL6102 | Expression |
| T1D | 5 | GSE30208 | 37 | 26 | GPL6102 | Expression |
| T1D | 6 | GSE30210 | 125 | 122 | GPL6947 | Expression |
| T1D | 7 | GSE33440 | 16 | 6 | GPL6947 | Expression |
| T1D | 8 | GSE35725 | 70 | 44 | GPL570 | Expression |
| T1D | 9 | GSE43488 | 187 | 169 | GPL13667 | Expression |
| T1D | 10 | GSE44314 | 10 | 6 | GPL6480 | Expression |
| MS | 1 | GSE40360 | 28 | 19 | GPL13534 | Mehtylation |
| MS | 2 | GSE17846 | 20 | 21 | GPL9040 | microRNA |
| MS | 3 | GSE21079 | 59 | 37 | GPL8178 | microRNA |
| MS | 4 | GSE27690 | 4 | 5 | GPL10850 | microRNA |
| MS | 5 | GSE31568 | 23 | 20 | GPL9040 | microRNA |
| MS | 6 | GSE39643 | 8 | 8 | GPL15847 | microRNA |
| MS | 7 | GSE43590 | 11 | 9 | GPL14613 | microRNA |
| MS | 8 | GSE3447 | 4 | 8 | GPL538 | Expression |
| MS | 9 | GSE5839 | 3 | 1 | GPL96 | Expression |
| MS | 10 | GSE10064 | 6 | 4 | GPL2895 | Expression |
| MS | 11 | GSE14895 | 40 | 24 | GPL96,GPL571 | Expression |
| MS | 12 | GSE16461 | 8 | 8 | GPL570 | Expression |
| MS | 13 | GSE17048 | 99 | 45 | GPL6947 | Expression |
| MS | 14 | GSE17393 | 8 | 7 | GPL571 | Expression |
| MS | 15 | GSE21942 | 14 | 15 | GPL570 | Expression |
| MS | 16 | GSE23832 | 8 | 4 | GPL6244 | Expression |
| MS | 17 | GSE26484 | 6 | 4 | GPL570 | Expression |
| MS | 18 | GSE27688 | 18 | 12 | GPL6480 | Expression |
| MS | 19 | GSE32645 | 3 | 3 | GPL4133 | Expression |
| MS | 20 | GSE32915 | 12 | 4 | GPL4133 | Expression |
| MS | 21 | GSE38010 | 5 | 2 | GPL570 | Expression |
| MS | 22 | GSE41890 | 44 | 24 | GPL6244 | Expression |
| MS | 23 | GSE43591 | 10 | 10 | GPL570 | Expression |
| RA | 1 | GSE19033 | 5 | 15 | GPL9183 | Mehtylation |
| RA | 2 | GSE42861 | 354 | 335 | GPL13534 | Mehtylation |
| RA | 3 | GSE21118 | 2 | 1 | GPL10268 | microRNA |
| RA | 4 | GSE34391 | 1 | 1 | GPL11487 | microRNA |
| RA | 5 | GSE37425 | 4 | 2 | GPL15467 | microRNA |
| RA | 6 | GSE46012 | 3 | 3 | GPL11316 | microRNA |
| RA | 7 | GSE50646 | 24 | 8 | GPL16770 | microRNA |
| RA | 8 | GSE1919 | 5 | 5 | GPL91 | Expression |
| RA | 9 | GSE2053 | 4 | 4 | GPL1740 | Expression |
| RA | 10 | GSE4255 | 1 | 1 | GPL3463 | Expression |
| RA | 11 | GSE4588 | 15 | 19 | GPL570 | Expression |
| RA | 12 | GSE10024 | 2 | 4 | GPL96 | Expression |
| RA | 13 | GSE10500 | 5 | 3 | GPL8300 | Expression |
| RA | 14 | GSE12021 | 24 | 13 | GPL96,GPL97 | Expression |
| RA | 15 | GSE13026 | 24 | 21 | GPL5215 | Expression |
| RA | 16 | GSE15573 | 18 | 15 | GPL6102 | Expression |
| RA | 17 | GSE17755 | 118 | 53 | GPL1291 | Expression |
| RA | 18 | GSE23561 | 6 | 9 | GPL10775 | Expression |
| RA | 19 | GSE24060 | 6 | 60 | GPL7264 | Expression |
| RA | 20 | GSE29746 | 9 | 11 | GPL4133 | Expression |
| RA | 21 | GSE49604 | 6 | 2 | GPL8432,GPL10558 | Expression |
| RA | 22 | GSE55235 | 10 | 10 | GPL96 | Expression |
| RA | 23 | GSE55457 | 13 | 10 | GPL96 | Expression |
| SLE | 1 | GSE19033 | 5 | 15 | GPL9183 | Mehtylation |
| SLE | 2 | GSE27895 | 11 | 12 | GPL8490 | Mehtylation |
| SLE | 3 | GSE34391 | 1 | 1 | GPL11487 | microRNA |
| SLE | 4 | GSE37426 | 8 | 4 | GPL15468 | microRNA |
| SLE | 5 | GSE4588 | 15 | 19 | GPL570 | Expression |
| SLE | 6 | GSE8650 | 76 | 42 | GPL96,GPL97 | Expression |
| SLE | 7 | GSE10325 | 39 | 28 | GPL96 | Expression |
| SLE | 8 | GSE12374 | 11 | 6 | GPL1291 | Expression |
| SLE | 9 | GSE17755 | 22 | 53 | GPL1291 | Expression |
| SLE | 10 | GSE20864 | 21 | 45 | GPL1291 | Expression |
| SLE | 11 | GSE21649 | 4 | 4 | GPL10381 | Expression |
| SLE | 12 | GSE22098 | 110 | 81 | GPL6947 | Expression |
| SLE | 13 | GSE22132 | 1 | 1 | GPL570 | Expression |
| SLE | 14 | GSE24060 | 6 | 18 | GPL7264 | Expression |
| SLE | 15 | GSE29536 | 96 | 108 | GPL6102 | Expression |
| SLE | 16 | GSE30153 | 17 | 9 | GPL570 | Expression |
| SLE | 17 | GSE50395 | 3 | 3 | GPL4133 | Expression |
| SLE | 18 | GSE50635 | 33 | 16 | GPL6244 | Expression |

**Supplementary Table 2: Significant differences of the number of genes on four diseases**

| GSE_number | number_of_different_gene |
| --- | --- |
| DBI_GSE24147 | 0 |
| DBI_GSE29142 | 0 |
| DBI_GSE30208 | 47 |
| DBI_GSE30209 | 8 |
| DBI_GSE30210 | 25 |
| DBI_GSE33440 | 40 |
| DBI_GSE35725 | 2 |
| DBI_GSE43488 | 124 |
| DBI_GSE44314 | 0 |
| DBI_GSE56606 | 0 |
| MS_GSE10064 | 0 |
| MS_GSE14895-GPL571 | 972 |
| MS_GSE14895-GPL96 | 0 |
| MS_GSE16461 | 0 |
| MS_GSE17048 | 3 |
| MS_GSE17393 | 0 |
| MS_GSE21942 | 588 |
| MS_GSE23832 | 0 |
| MS_GSE26484 | 0 |
| MS_GSE27688 | 0 |
| MS_GSE32645 | 0 |
| MS_GSE32915 | 0 |
| MS_GSE3447 | 43 |
| MS_GSE38010 | 0 |
| MS_GSE41890 | 0 |
| MS_GSE43591 | 110 |
| MS_GSE5839 | 629 |
| MS_GSE40360 | 1 |
| MS_GSE17846 | 1695 |
| MS_GSE21079 | 0 |
| MS_GSE27690 | 0 |
| MS_GSE31568 | 434 |
| MS_GSE39643 | 1051 |
| MS_GSE43590 | 0 |
| RA_GSE10024 | 0 |
| RA_GSE10500 | 5 |
| RA_GSE12021-GPL96 | 0 |
| RA_GSE12021-GPL97 | 0 |
| RA_GSE13026 | 218 |
| RA_GSE15573 | 215 |
| RA_GSE17755 | 484 |
| RA_GSE1919 | 0 |
| RA_GSE2053 | 0 |
| RA_GSE23561 | 11 |
| RA_GSE24060 | 0 |
| RA_GSE29746 | 0 |
| RA_GSE4255 | 1245 |
| RA_GSE4588 | 377 |
| RA_GSE49604-GPL10558 | 0 |
| RA_GSE49604-GPL8432 | 0 |
| RA_GSE55235 | 821 |
| RA_GSE55457 | 86 |
| RA_GSE19033 | 4 |
| RA_GSE42861 | 1354 |
| RA_GSE21118 | 478 |
| RA_GSE34391 | 0 |
| RA_GSE37425 | 0 |
| RA_GSE46012 | 0 |
| RA_GSE50646 | 0 |
| SLE_GSE10325 | 9 |
| SLE_GSE12374 | 14 |
| SLE_GSE17755 | 1284 |
| SLE_GSE20864 | 1262 |
| SLE_GSE21649 | 10 |
| SLE_GSE22098 | 83 |
| SLE_GSE22132 | 1436 |
| SLE_GSE24060 | 0 |
| SLE_GSE29536 | 98 |
| SLE_GSE30153 | 0 |
| SLE_GSE4588 | 91 |
| SLE_GSE50395 | 0 |
| SLE_GSE50635 | 110 |
| SLE_GSE8650-GPL96 | 969 |
| SLE_GSE8650-GPL97 | 551 |
| SLE_GSE19033 | 22 |
| SLE_GSE27895 | 0 |
| SLE_GSE34391 | 0 |
| SLE_GSE37426 | 0 |
